# Psychological impact of climate change emergency: an attempt to define eco-anxiety

**DOI:** 10.3389/fpsyg.2024.1375803

**Published:** 2024-09-17

**Authors:** Luisa Orrù, Stefania Mannarini

**Affiliations:** ^1^Department of Philosophy, Sociology, Education, and Applied Psychology, Section of Applied Psychology, University of Padua, Padua, Italy; ^2^Center for Intervention and Research on Family Studies (CIRF), Department of Philosophy, Sociology, Education, and Applied Psychology, Section of Applied Psychology, University of Padova, Padua, Italy

**Keywords:** eco-anxiety, mental health, climate change, emotional regulation, climate change anxiety

## 1 Introduction

Climate change was an important emergency of our time. Climate change touched on existential issues: hope for the global future (Ojala, 2021), political (Holthaus, 2022) issues that involved conflicts of various kinds, different coping measures (Håkansson et al., 2019), children's education (Hadar et al., 2020; Pihkala, 2020a), impacts on human health (Thoma et al., 2021) and different clinical and psychotherapeutic approaches (Raile, 2023; Doherty et al., 2022). Efforts to combine psychological and social studies in relation to the ecological and climate crisis have increased since 2010 (Hoggett, 2019). As the impact on our planet becomes increasingly felt, the results on mental health (Turchi et al., 2022, 2023) were becoming more apparent (Manning and Clayton, 2018; Dodgen et al., 2016; Doherty and Clayton, 2011). In addition to post-traumatic stress disorder following a catastrophic event, anxiety and depression were also diagnosed in the absence of a specific event; so much so that there was a specific declination of these two clinical frameworks emerged with related measurement scales, such as the Climate Change Anxiety Scale (Clayton and Karazsia, 2020), the Climate Change Worry Scale (Stewart, 2021) and the Eco-Anxiety scale (Hogg et al., 2021). Literature data suggested a worse mental health in combination with ecological anxiety and functional compromise, anxiety, stress, insomnia, and depression. In young adults and women, correlations are founded with cognitive and emotional symptoms, doubts about the future and unwillingness to become parents. There were various symptoms analyzed to qualify for eco-anxiety, which may referred to different levels of eco-anxiety such as anxiety, fear, grief, worry and despair. In the wide range of eco-anxiety definitions used in the different studies (Boluda-Verdu et al., 2022), most of them, as an implication to climate change, considered eco-anxiety like a complex of different emotions: helpless, powerless, sad, depressed, angry (Helm et al., 2021; Searle and Gow, 2010), feeling anxious (Helm et al., 2021; Stanley et al., 2021), feeling or being worried (Berry and Peel, 2015; Ogunbode et al., 2021; Sciberras and Fernando, 2022; Searle and Gow, 2010), feeling tense (Ogunbode et al., 2021; Searle and Gow, 2010), sadness, guilt (Helm et al., 2021), fear (Helm et al., 2021; Stanley et al., 2021), anxiety (Ogunbode et al., 2021), and habitual worrying (Verplanken et al., 2020). In other studies (Patrick et al., 2022; Schwartz et al., 2022), eco-anxiety was analyzed as functional impairment and as cognitive-emotional impairment. Ecological anxiety has been associated with the behavior of those who choose to act pro-environmentally (Verplanken et al., 2020). Climate activism, in those with symptoms of major depression, was an important component in mitigating the emotional and cognitive impact of climate anxiety (Schwartz et al., 2022), although Stanley et al. (2021) found that eco-anxiety is associated with reduced community action. Finally, a number of studies (Helm et al., 2021; Schneider-Mayerson and Leong, 2020) pointed out that climate anxiety may be an important component of future planning and willingness to become parents (Patrick et al., 2022). In line with the studies described above, the research question could be “was there a need for a common definition of this construct?” And “were there any other effects of this clinical configuration that need to be taken into account?”

## 2 Discussion

### 2.1 Definitions of eco-anxiety construct

The term “eco-anxiety” is accepted into the lexicon of the APA (2018). In the American Psychological Association's 2018 “Stress in America” survey, 51% of respondents identified climate change as “a somewhat or significant source of stress” (APA, 2018): it is concern about climate change combined with concern about the future. The term eco-anxiety was coined by Glenn Albrecht who defines it as a chronic fear of environmental doom (Coffey et al., 2021; Clayton, 2017; Inauen et al., 2021). Uncertainty is highlighted regarding the eco-anxiety construct (Coffey et al., 2021): there were more than ten distinct operationalizations of this notion in the existing literature. A widely cited definition of eco-anxiety was: “the generalized sense that the ecological foundations of existence are in the process of collapse” (Albrecht, 2012, p. 241–264). Some scholars use eco-anxiety as a synonym for climate-anxiety, while others like to treat the two terms separately (Pihkala, 2020b). With Innocenti (2022, p. 77) eco-anxiety encompassed an emotional experience that was very common in the everyday life of Western society: “anxiety, that emotion of apprehension and worry that something harmful, threatening, and terrible might happen at any moment, without having the possibility to control or foresee it.” This state of anxiety could also be directed toward the natural environment, when a person has the constant perception that something terrible and irreparable is undermining the ecological integrity of their planet. In psychology the term “eco-anxiety” is used to refer to subclinical forms of anxiety, guilt and depression aroused by the thought of climate change and other environmental aspects. Kidner (2014) claims that the loss of a sense of security caused by progressive environmental degradation has been underestimated by mainstream scientific approaches. Other commentators have defined the lack of initiative to protect the environment as “apathy,” but psychologists such as Randall (2009) and Lertzman (2010) argued that this strange paralysis was a “freezing” response to scale of the problem. In recent literature, eco-anxiety was a word used to refer to the experience of anxiety related to environmental crises (Pihkala, 2020b; Hickman, 2020), and as Hogg et al. (2021, p. 1) said “given the interconnectedness of environmental issues in our global ecosystem, and evidence that people report anxiety about other types of environmental problems, it is unclear whether climate change anxiety is distinct from other types of environmental anxiety.” He concludes: “Despite its growing research significance, there is still limited understanding of what the psychological experience of eco-anxiety entails” (Hogg et al., 2021, p. 1). Raile (2023) highlighted that eco-anxiety is usually described as an adequate and non-pathological reaction to climate change. But psychotherapeutic treatment may be recommended if life projects or quality of life are at risk. According with Raile (2023), psychotherapeutic treatment was useful and it was necessary to define at least which was the syndromic framework, even if eco-anxiety was not a pathological reaction (Campolonghi and Orrù, 2023). This point could be important for positioning the treatment of eco-anxiety. The literature showed that eco-anxiety was not always associated with anxiety but is often associated with depression; it is considered as an emotional problem and it is a collection of different emotional states (Thoma et al., 2021; Pihkala, 2020b). Therefore, to plan the treatment of eco-anxiety it could be important to analyse not only the framework with which it is associated, but also the risk factors to understand interindividual differences in the response and adaptation to climate crisis. Given the emergency of the climate crisis and the urgency to offer direction for clinicians, in accordance with Thoma et al. (2021, p. 16) “it is of crucial importance that future research examines this neglected relationship (between mental health and climate and environmental crisis) in light of the identified processes and pathways, including the consideration of potential vulnerability and protective factors.”

### 2.2 Assessing eco-anxiety

As Hwong et al. (2022) pointed out, there are gaps in the research on climate change and mental health: different constructs definitions, reference theories, study designs, data collection methods, and analyses. Considering this, what has emerged was preliminary and therefore needs to be verified (Mannarini and Boffo, 2013; Mannarini, 2009). It would be appropriate to converge at least on appropriate and validated rating scales to measure levels of eco-anxiety. When discussing the measurement of eco-anxiety, some authors used Clayton (2020)'s scale (defined to climate change anxiety). However, there was a problem: eco-anxiety and climate change anxiety were different constructs. In fact, it was unclear whether climate change anxiety was different from other types of environmental anxiety: it was possible that eco-anxiety captured anxiety in response to the global environmental crisis, unlike existing scales that specifically measured climate change anxiety. This construct appeared to be broader than climate change anxiety (Hogg et al., 2021). Clayton and Karazsia (2020) examined the factorial structure of their climate change anxiety scale, and suggested a four-factor structure, they assessed depression and anxiety using a four-item measure that combined anxiety and depression into a single sum score. Mouguiama-Daouda et al. (2022, p. 125) write about uncertainty in the very structure of the CAS. They conclude that “given the theoretical and clinical relevance of improving our understanding of the potential interplay between, on the one hand, climate anxiety and, on the other hand, general anxiety and depression (e.g., Clayton, 2020), such an absence of consideration for the distinction between anxiety and depression is problematic and deserves a more careful audit.” Such a lack of attention to the distinction between them was problematic and deserves more careful scrutiny. When analyzing anxiety and depression separately, it was found that all factors showed positive correlations with depression, but not with anxiety. Therefore, since previous studies have not analyzed the two constructs separately, thus it was possible that climate change anxiety is primarily associated with depression and not anxiety. They also agreed with Wullenkord et al. (2021), who said that the CAS did not capture climate anxiety, rather than an emotional impairment. According with Clayton's (2020) hypothesis about CCA as a complex psychological response associated with emotions. There was not a single definition, and the scales have not helped to clarify the condition. Hogg et al. (2021) proposed a four-factor, 13-item scale to specifically assess eco-anxiety. The authors write that eco-anxiety was not a pathological disease or clinical disorder, further supporting the distinction between eco-anxiety and general anxiety: eco-anxiety went beyond affective symptoms. From what has been reported so far, it appeared that the various operationalizations have in common a definition of eco-anxiety as an emotional (as well as cognitive and functional) state. Ágoston et al. (2022) proposed an Eco-Anxiety Questionnaire (EAQ-22) consisted of two factors: habitual ecological worry and the negative consequences of eco-anxiety. The study showed that eco-anxiety, eco-guilt, and eco-grief are multifactorial constructs, in line with literature. Some results are partially in line with the results of the current studies: changes in the weather was less important, and conflicts with others did not appear directly connect with eco-anxiety in other studies. Furthermore, Micoulaud-Franchi et al. (2024), validated the French EAQ-22 as an in-depth tool for assessing eco-anxiety in line with Wakefield and Conrad (2020)'s analysis that support the disorder includes a naturalistic component of dysfunction (failure of biologically designed functioning) and a value (harm) component, both of which are required for disorder attributions.

However, it might be useful to conduct further research that explores precisely the relationship between emotion management tasks associated with an emotional state related to climate change and the harm component. Indeed, it might be useful to explore whether there is a causal or correlational association between eco-anxiety and emotional regulation (Ejelöv et al., 2018; Ojala, 2012). To determine the level of distress caused by eco-anxiety, we considered a central role of emotional regulation (Gross, 1998, 2013; Rossi et al., 2022; Manning and Clayton, 2018), as that process through which individuals influence which emotion they have, when they feel it, and how they experience it. According to Hughes et al. (2020) and McRae and Gross (2020), emotional regulation become increasingly important in psychological health studies as an essential characteristic of good functioning (Gross and Muñoz, 1995). Emotion regulation is defined concerning competences to understand and regulate emotions (Gratz and Roemer, 2004; Gross and Muñoz, 1995). When these competences are lacking, we referred to it as emotion dysregulation (Gratz and Roemer, 2004). Therefore, research by Zlomke and Hahn (2010) and Salters-Pedneault et al. (2006) has shown that cognitive emotion regulation strategies play an important role in the management of anxiety. If many studies available in the literature focused on the construct of coping (which, despite having similarities with the process of emotional regulation, is not comparable to it), this contribution aimed to focus attention on the emotional part that influences in managing eco-anxiety. In fact, if this were the case, then clinical treatment could shift from treating the anxious and/or depressive state to modifying the emotional management of climate change, helping the patient to implement more effective strategies of emotional regulation, defining goals for managing one's emotions, as well as defining an active and proactive role in dealing with climate change (Balottin et al., 2017; Mannarini et al., 2013). This possibility here, therefore, would allow clinicians to shift their focus from the definition of causes (past traumas, dysfunctional bonds, etc.) to the construction of adequate and relevant ways of dealing with uncertainty toward the future and the emotions it brings with it, precisely in order to preserve quality of life and future planning, which eco-anxiety seemed to put at risk.

## 3 Conclusion

The literature on the impact of climate change on mental health and on eco-anxiety in particular highlighted some research questions: was it necessary to measure each symptom of this construct separately, as recent systematic reviews have underlined (Boluda-Verdu et al., 2022)? Then, there were found more than ten distinct operationalizations of this notion in the existing literature: was there an urgent need for a common definition of this construct that can be operationalised and measured in a single and globally recognized way? Not all the authors agreed whether, when we analyzed eco-anxiety, we were looking at a disorder, a syndromic framework or a non-clinical condition. Many authors wrote about eco-anxiety as an emotional response to a (real or at least perceived) stressful situation. Did climate change affect emotion regulation (Ejelöv et al., 2018; Ojala, 2012)? If emotion regulation was a good way to treat eco-anxiety, it could be useful to analyse the link between eco-anxiety and emotion regulation in the clinical setting to define specific treatment indications. In light of the above, the question arised as to how to find a clinical placement for this phenomenon, despite the fact eco-anxiety was not yet considered a recognized anxiety or depressive disorder, but rather an understandable response to the severity of the ecological crisis, although there were obvious cases where eco-anxiety was strong enough to require mental health support (Doherty, 2016; Manning and Clayton, 2018; Pihkala, 2019). Here, if we considered eco-anxiety as an emotion/set of overwhelming emotions that could affect the quality of life, although it did not constitute a pathological clinical configuration (Pihkala, 2020b), then it was possible to consider that clinical treatment should focus on emotion regulation. It could be useful to consider that, in the presence of eco-anxiety, it was useful for the clinician to explore the patient's competences to regulate emotions, as a possible risk factor, in addition to the presence of other previous clinical configuration on which eco-anxiety is grafted (e.g., childhood trauma, generalized anxiety, and others), as Raile (2023) described through the clinical cases illustrated in his contribution. In line with these, eco-anxiety may be configured as the field of application of the treatment but not the objective.
